# Supplementary figures and images for: Projected changes of alpine grassland carbon dynamics in response to climate change and elevated CO2 concentrations under Representative Concentration Pathways (RCP) scenarios
Source: PLoS One. 2019 Jul 22;14(7):e0215261. doi: 10.1371/journal.pone.0215261 (PMC6645462; doi:10.1371/journal.pone.0215261)

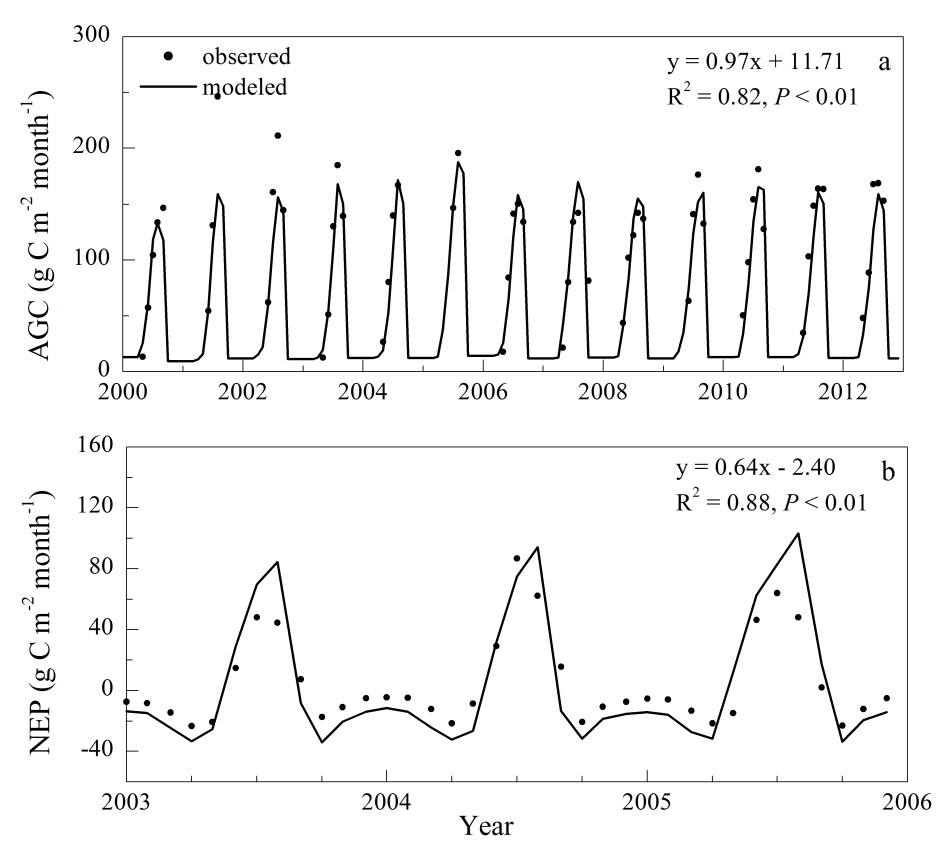

Supplement: S1 Fig — Validation of observed and modeled aboveground biomass (a), and net ecosystem production (b) at the Haibei research station of the Tibetan Plateau. (TIF) [file pone.0215261.s001.tif]

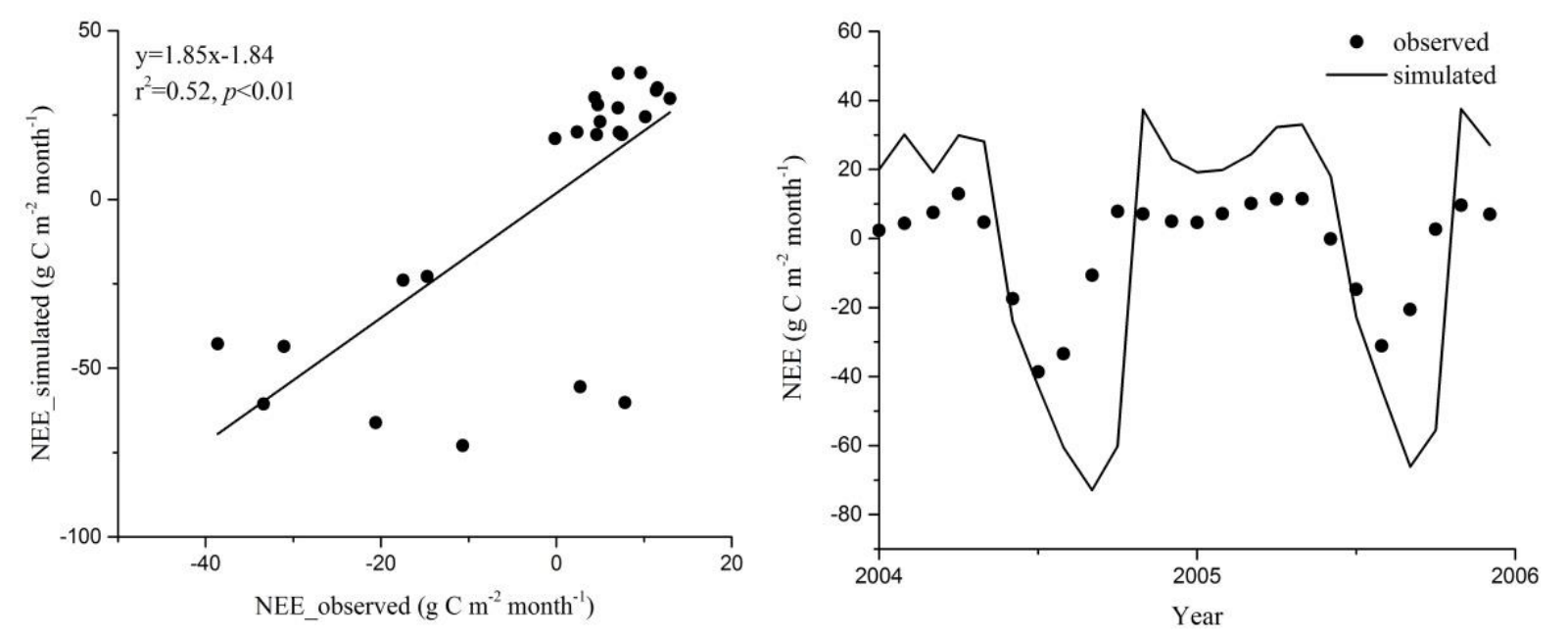

Supplement: S2 Fig — (TIF) [file pone.0215261.s002.tif]

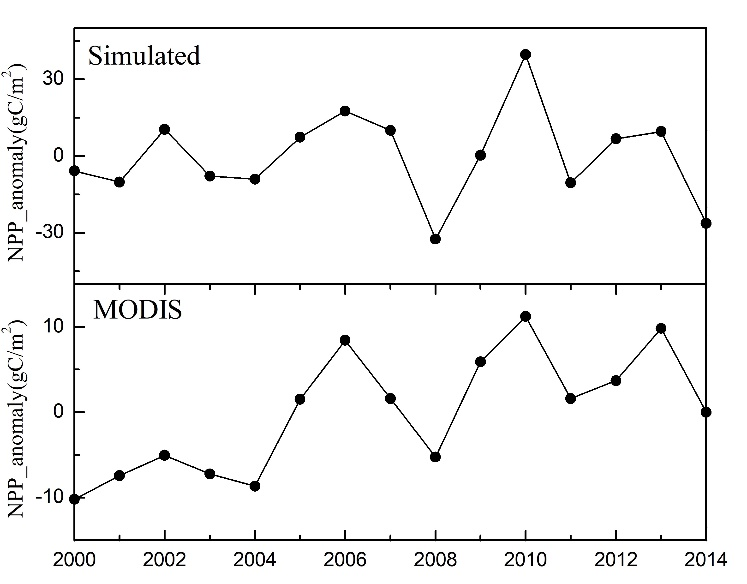

Supplement: S3 Fig — (TIF) [file pone.0215261.s003.tif]
