# Supplementary material for: Projected changes of alpine grassland carbon dynamics in response to climate change and elevated CO2 concentrations under Representative Concentration Pathways (RCP) scenarios
Source: PLoS One. 2019 Jul 22;14(7):e0215261. doi: 10.1371/journal.pone.0215261 (PMC6645462; doi:10.1371/journal.pone.0215261)
Supplement: S1 Table — (DOCX) [file pone.0215261.s004.docx]

**S1 Table. Comparisons of carbon flux in the Tibetan Plateau grassland between the CENTURY simulation and previous studies [**[**1**](#_ENREF_1)**]**

| Total NPP (Pg C yr^-1^) | Area (10^6^ km^2^) | NPP  (g C m^-2^ yr^-1^) | Model | Study period | Reference |
| --- | --- | --- | --- | --- | --- |
| 0.1 | 0.9 | 127.5 | CASA | 1982-1999 | [[7](#_ENREF_7)] |
| 0.2 | 1.5 | 120.8 | CASA | 1982-2009 | [[2](#_ENREF_2)] |
| 0.3 | 1.2 | 282.7 | TEM | 1990s | [[5](#_ENREF_5)] |
| 0.5 | 1.3 | 340.8 | Satellite-based statistical model | 1982-1999 | [[3](#_ENREF_3)] |
| 0.3 | 1.4 | 233.0 | ORCHIDEE | 1980-1990 | [[8](#_ENREF_8)] |
| 0.3 | 1.4 | 244.7 | CENTURY | 1901-2010 | This study |
| 0.4 | 1.4 | 259.3 | CENTURY | 1981-2010 | This study |
| Total NEP (Tg C yr^-1^) | Area (10^6^ km^2^) | NEP  (g C m^-2^ yr^-1^) | Model | Study period | Reference |
| 17.6 | NA | NA | Inventory-satellite-based estimation | 1980s-1990s | [[9](#_ENREF_9)] |
| 11.8 | 1.4 | 8.3 | ORCHIDEE | 1961-2009 | [[10](#_ENREF_10)] |
| 29.8 | 1.2 | 24.2 | TEM | 1990s | [[5](#_ENREF_5)] |
| 7.4 | 1.4 | 5.3 | CENTURY | 1901-2010 | This study |
| 14.1 | 1.4 | 10.1 | CENTURY | 1981-2010 | This study |

# References

1. Lin X, Han P, Zhang W, Wang G (2017) Sensitivity of alpine grassland carbon balance to interannual variability in climate and atmospheric CO2 on the Tibetan Plateau during the last century. Global and Planetary Change 154: 23-32.

2. Zhang Y, Wei Q, Zhou C, Ding M, Liu L, Gao J, et al. (2014) Spatial and temporal variability in the net primary production of alpine grassland on the Tibetan Plateau since 1982. Journal of Geographical Sciences 24: 269-287.

3. Piao S, Fang J, Zhou L, Tan K, Tao S (2007) Changes in biomass carbon stocks in China's grasslands between 1982 and 1999. Global biogeochemical cycles 21: doi:10.1029/2005GB002634.

4. Pan Y, Birdsey R, Hom J, Mccullough K, Clark K (2006) Improved estimates of net primary productivity from modis satellite data at regional and local scales. Ecological Applications 16: 125-132.

5. Zhuang Q, He J, Lu Y, Ji L, Xiao J, Luo T (2010) Carbon dynamics of terrestrial ecosystems on the Tibetan Plateau during the 20th century: an analysis with a process‐based biogeochemical model. Global Ecology and Biogeography 19: 649-662.

6. Pan S, Dangal SRS, Tao B, Yang J, Tian H (2015) Recent patterns of terrestrial net primary production in Africa influenced by multiple environmental changes. Ecosystem Health and Sustainability 1: 1-15.

7. Piao S, Fang J, He J (2006) Variations in vegetation net primary production in the Qinghai-Xizang Plateau, China, from 1982 to 1999. Climatic Change 74: 253-267.

8. Tan K, Ciais P, Piao S, Wu X, Tang Y, Vuichard N, et al. (2010) Application of the ORCHIDEE global vegetation model to evaluate biomass and soil carbon stocks of Qinghai-Tibetan grasslands. Global Biogeochemical Cycles 24: doi:10.1029/2009GB003530.

9. Piao S, Fang J, Ciais P, Peylin P, Huang Y, Sitch S, et al. (2009) The carbon balance of terrestrial ecosystems in China. Nature 458: 1009-1013.

10. Piao S, Tan K, Nan H, Ciais P, Fang J, Wang T, et al. (2012) Impacts of climate and CO 2 changes on the vegetation growth and carbon balance of Qinghai–Tibetan grasslands over the past five decades. Global and Planetary Change 98: 73-80.
