# Supplementary material for: Projected changes of alpine grassland carbon dynamics in response to climate change and elevated CO2 concentrations under Representative Concentration Pathways (RCP) scenarios
Source: PLoS One. 2019 Jul 22;14(7):e0215261. doi: 10.1371/journal.pone.0215261 (PMC6645462; doi:10.1371/journal.pone.0215261)
Supplement: S1 File — (PDF) [file pone.0215261.s005.pdf]

Supporting information for online publication only:

Projected changes of alpine grassland carbon dynamics in response to climate change and elevated CO_2 concentrations under Representative Concentration Pathways (RCP) scenarios

Pengfei Han[1¶*], Xiaohui Lin[2¶], Wen Zhang[2*], Guocheng Wang[2], Yinan Wang[3]

[1]State Key Laboratory of Numerical Modeling for Atmospheric Sciences and Geophysical Fluid Dynamics, Institute of Atmospheric Physics, Chinese Academy of Sciences, Beijing 100029, China

[2]State Key Laboratory of Atmospheric Boundary Layer Physics and Atmospheric Chemistry, Institute of Atmospheric Physics, Chinese Academy of Sciences, Beijing 100029, China

[3]Laboratory of Middle Atmosphere and Global Environment Observation, Institute of Atmospheric Physics, Chinese Academy of Sciences, Beijing 100029, China

[*]**Correspondence**: pfhan@mail.iap.ac.cn; zhw@mail.iap.ac.cn

[¶]These authors contributed equally to this work.

Tel.: +86-10-8299-5368

Model validations and comparisons with other studies

Field sampling and flux tower observed data from the Haibei research station (37°29'-37°45'N, 101°12'-101°23'E) in the northeastern part of the Qinghai-Tibetan Plateau were used to validate the performance of the parameterized CENTURY model [1]. In this study, the daily observed net ecosystem exchange (NEE) during the period of 2003-2005 and monthly observed aboveground biomass (AGC) during the period of 2000-2012 were selected to validate the simulated results. As shown in S1 Fig., the simulated monthly AGC exhibited a good agreement with the observed data with a slope of 0.97, and an intercept were less than 20 g C m^{-2} (R^2= 0.82, P < 0.01, S1a Fig.). Compared to the eddy-covariance flux data, the model was capable of capturing the variations of NEE with a slope of 0.64, and an intercept of 2.40 g C m^{-2} (R^2= 0.88, P < 0.01, S1b Fig.). The observed AGC and growing season temperature (GST = 9.1 ℃) in 2005 was higher than in other years (7.1 ℃ ~ 8.9 ℃). Additionally, the multiyear annual precipitation value of 413.5 mm was used as the 2003 value, because the observed annual rainfall of 11.5 mm in 2003 was much lower than in other years (352.6 ~ 520.0 mm). Consequently, the seasonal amplitude of the simulated net ecosystem production (NEP) in 2003 and 2005 was slightly higher than that derived from the flux tower data. Overall, we found that the parameterized CENTURY model was capable to simulate the carbon fluxes of the alpine grassland.

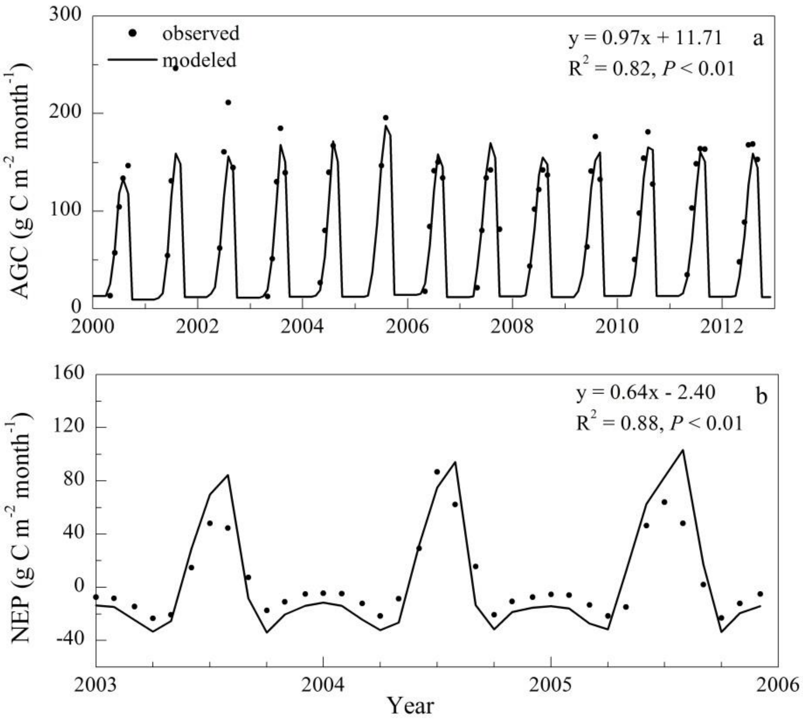

S1 Fig. Validation of observed and modeled aboveground biomass (a), and net ecosystem production (b) at the Haibei research station of the Tibetan Plateau [1]

The Chinese Ecosystem Research Network (CERN) and ChinaFlux network have performed comprehensive observations on carbon budgets at major ecosystems of China. On TP, there were two eddy-covariance observation sites of meadow grassland: Damxung and Naqu, which measured net ecosystem exchange (NEE). However, they provide carbon exchange measurements without sufficient information of other variables (i.e., the nutrient contents of C, N, and P in the plant material and soil) to drive the CENTURY model. We tried to make use of the NEE observations at Damxung, which has observation of NEE from 2004 to 2005, to further validate the calibrated CENTURY (S1 Fig.). The simulated NEE at Damxung agreed well with the observational fluxes (r^2=0.52, p<0.01). Similar results were also found by Zhuang et

al. (2010). We suggest that field studies to incorporate more extensive field measurements including climate, vegetation, soil and NEE, and collaborate and share observed data with modelers (Jin et al. 2015).

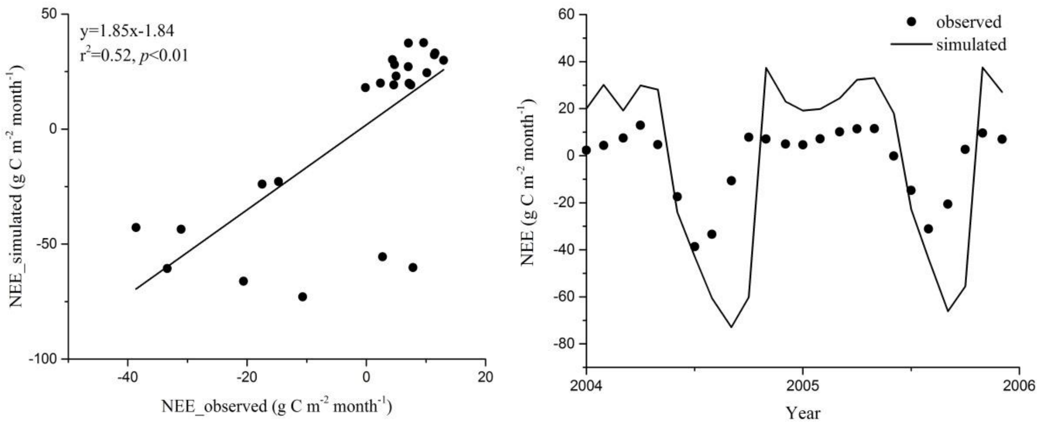

S2 Fig. Validation of observed and modeled net ecosystem exchange at the Damxung research station of the Tibetan Plateau.

Unfortunately, we didn’t find a site in steppe grassland of TP that had necessary data for the CENTURY model’s calibration. Instead, we compared the modeling results with the Moderate Resolution Imaging Spectroradiometer (MODIS) NPP products (MOD17A3) during 2000-2014 on the regional scale (S3 Fig.). The simulated NPP had a similar trend with MODIS derived NPP, both of which had a notable increase in 2010, and a sudden decrease in 2008.

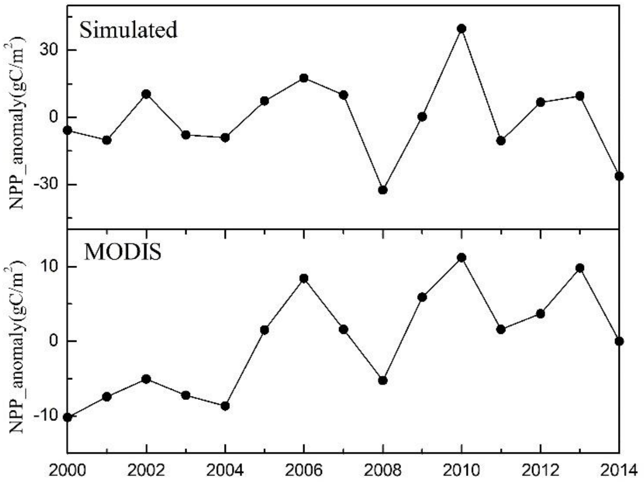

S3 Fig. Comparison of NPP anomaly between simulated results (CENTURY) and satellite based products of MODIS NPP (MOD17A3).

At the regional scale, we also conducted a comprehensive comparison analysis between this study and existing publications (Table S1), showing that the current simulated results were within the ranges of previous estimations driven by different models [1]. In this study, the simulated annual NPP of the Tibetan Plateau grassland over the period from 1901 to 2010 was 244.6 g C m^{-2} yr^{-1}, and was 259.3 g C m^{-2} yr^{-1} during the period 1981 ~ 2010, which were comparable to other estimates (e.g., 120.8 ~ 340.8 g C m^{-2} yr^{-1}) [2,3]. The low NPP value simulated by the CASA model was probably attributed to the light utilization efficiency (ε), which is constrained by low temperature of the alpine climate of the Tibetan Plateau [1,4]. The limited observational data used in the parameterization, leading to a slightly high NPP simulated by the TEM model (282.7 g C m^{-2} yr^{-1}) [5]. Additionally, due to the lack of detailed soil and vegetation information, the satellite-based model may not be sufficiently accurate to estimate NPP by imprecise parameterization [4,6].

S1 Table. Comparisons of carbon flux in the Tibetan Plateau grassland between the CENTURY simulation and previous studies [1]

Total NPP (Pg C yr^{-1})	Area (10^6 km^2)	NPP (g C m^{-2} yr^{-1})	Model	Study period	Reference
0.1	0.9	127.5	CASA	1982-1999	[7]
0.2	1.5	120.8	CASA	1982-2009	[2]
0.3	1.2	282.7	TEM	1990s	[5]
0.5	1.3	340.8	Satellite-based statistical model	1982-1999	[3]
0.3	1.4	233.0	ORCHIDEE	1980-1990	[8]
0.3	1.4	244.7	CENTURY	1901-2010	This study
0.4	1.4	259.3	CENTURY	1981-2010	This study

Total NEP (Tg C yr^{-1})	Area (10^6 km^2)	NEP (g C m^{-2} yr^{-1})	Model	Study period	Reference
17.6	NA	NA	Inventory-satellite-based estimation	1980s-1990s	[9]
11.8	1.4	8.3	ORCHIDEE	1961-2009	[10]
29.8	1.2	24.2	TEM	1990s	[5]
7.4	1.4	5.3	CENTURY	1901-2010	This study
14.1	1.4	10.1	CENTURY	1981-2010	This study

References

1. Lin X, Han P, Zhang W, Wang G (2017) Sensitivity of alpine grassland carbon balance to interannual variability in climate and atmospheric CO2 on the Tibetan Plateau during the last century. Global and Planetary Change 154: 23-32.
2. Zhang Y, Wei Q, Zhou C, Ding M, Liu L, Gao J, et al. (2014) Spatial and temporal variability in the net primary production of alpine grassland on the Tibetan Plateau since 1982. Journal of Geographical Sciences 24: 269-287.
3. Piao S, Fang J, Zhou L, Tan K, Tao S (2007) Changes in biomass carbon stocks in China's grasslands between 1982 and 1999. Global biogeochemical cycles 21: doi:10.1029/2005GB002634.
4. Pan Y, Birdsey R, Hom J, Mccullough K, Clark K (2006) Improved estimates of net primary productivity from modis satellite data at regional and local scales. Ecological Applications 16: 125-132.
5. Zhuang Q, He J, Lu Y, Ji L, Xiao J, Luo T (2010) Carbon dynamics of terrestrial ecosystems on the Tibetan Plateau during the 20th century: an analysis with a process - based biogeochemical model. Global Ecology and Biogeography 19: 649-662.
6. Pan S, Dangal SRS, Tao B, Yang J, Tian H (2015) Recent patterns of terrestrial net primary production in Africa influenced by multiple environmental changes. Ecosystem Health and Sustainability 1: 1-15.
7. Piao S, Fang J, He J (2006) Variations in vegetation net primary production in the Qinghai-Xizang Plateau, China, from 1982 to 1999. Climatic Change 74: 253-267.
8. Tan K, Ciais P, Piao S, Wu X, Tang Y, Vuichard N, et al. (2010) Application of the ORCHIDEE global vegetation model to evaluate biomass and soil carbon stocks of Qinghai-Tibetan grasslands. Global Biogeochemical Cycles 24: doi:10.1029/2009GB003530.
9. Piao S, Fang J, Ciais P, Peylin P, Huang Y, Sitch S, et al. (2009) The carbon balance of terrestrial ecosystems in China. Nature 458: 1009-1013.
10. Piao S, Tan K, Nan H, Ciais P, Fang J, Wang T, et al. (2012) Impacts of climate and CO 2 changes on the vegetation growth and carbon balance of Qinghai–Tibetan grasslands over the past five decades. Global and Planetary Change 98: 73-80.
